# Characterization of Microwave-Induced Electric Discharge Phenomena in Metal–Solvent Mixtures

**DOI:** 10.1002/open.201100013

**Published:** 2012-02-10

**Authors:** Wen Chen, Bernhard Gutmann, C Oliver Kappe

**Affiliations:** a Christian Doppler Laboratory for Microwave Chemistry (CDLMC), Institute of Chemistry, Karl-Franzens-University GrazHeinrichstrasse 28, 8010 Graz (Austria), Fax: (+43) (0)316-380-9840 E-mail: oliver.kappe@uni-graz.at; b On leave from the Shanghai Key Laboratory of Chemical Biology, East China University of Science and Technology130 Meilong Road, Shanghai, 200237 (P. R. China)

**Keywords:** arcing, electric discharges, magnesium, microwave chemistry, particle size, thermal decomposition

## Abstract

Electric discharge phenomena in metal–solvent mixtures are investigated utilizing a high field density, sealed-vessel, single-mode 2.45 GHz microwave reactor with a built-in camera. Particular emphasis is placed on studying the discharges exhibited by different metals (Mg, Zn, Cu, Fe, Ni) of varying particle sizes and morphologies in organic solvents (e.g., benzene) at different electric field strengths. Discharge phenomena for diamagnetic and paramagnetic metals (Mg, Zn, Cu) depend strongly on the size of the used particles. With small particles, short-lived corona discharges are observed that do not lead to a complete breakdown. Under high microwave power conditions or with large particles, however, bright sparks and arcs are experienced, often accompanied by solvent decomposition and formation of considerable amounts of graphitized material. Small ferromagnetic Fe and Ni powders (<40 μm) are heated very rapidly in benzene suspensions and start to glow in the microwave field, whereas larger particles exhibit extremely strong discharges. Electric discharges were also observed when Cu metal or other conductive materials such as silicon carbide were exposed to the microwave field in the absence of a solvent in an argon or nitrogen atmosphere.

## Introduction

High-speed microwave synthesis has attracted a considerable amount of attention in the past 25 years with new and innovative applications continuously being reported in the literature.^1^–^4^ In microwave chemistry, reaction times can often be reduced from days or hours to minutes or even seconds by efficient and rapid direct volumetric heating of the reaction mixture in a sealed vessel to temperatures far above the boiling point of the solvent under atmospheric conditions, typically to 300 °C/30 bar.^1^–^3^ In many instances, in part due to the exquisite control over the optimized reaction temperature and other important process parameters, microwave processing has been shown to dramatically reduce reaction times, but also to provide increased product yields and to enhance product purities compared with reactions that are conventionally heated at the reflux temperature of the solvent.^4^ These unique features explain the growing popularity of this nonclassical heating method in synthetic organic chemistry with currently more than 6000 publications, demonstrating the benefits of this technology.^1^, ^2^

In general, microwave chemistry relies on the ability of the reaction mixture to efficiently absorb microwave energy, taking advantage of microwave dielectric heating phenomena such as dipolar polarization or ionic conduction mechanisms.^1^, ^5^ This produces rapid internal heating (i.e., in-core volumetric heating) by direct interaction of electromagnetic irradiation with molecules, such as solvents, reagents and catalysts, that are present in the reaction mixture. Since the field of microwave-assisted organic synthesis is growing very rapidly, the number of applications that describe the use of metals as reagents or catalysts in such experiments has also increased significantly.^1^, ^2^, ^6^ Metals, being electric conductors, are known to strongly interact with microwave irradiation,^7^ and therefore, the possibility for electrostatic discharge phenomena (arcing) to occur under these conditions cannot be ignored.^8^ The situation is further aggravated by the fact that most microwave-assisted transformations today are performed in high field density, single-mode cavities that generally do not allow a visual inspection of the reaction vessel during the microwave irradiation process.^9^, ^10^ Therefore, most users would be unaware of potentially destructive and hazardous arcing phenomena that may occur under these conditions.

In recent publications, the microwave-assisted formation of Grignard reagents by insertion of Mg metal into the carbon–halogen bond of various aryl halides has been reported using both multi- and single-mode microwave reactors.^11^, ^12^ The experiments were performed at ∼65 °C using dry tetrahydrofuran (THF) as the solvent in an Ar atmosphere. The interaction between the microwave field and the Mg metal produced clearly visible and audible electric discharge phenomena between the Mg particles (arcing).^11^, ^12^ Applying a high field density, single-mode microwave reactor with a built-in camera, extremely violent arcing was observed, which led to the formation of significant amounts of carbonaceous material formed by the decomposition of THF.^12^ These results clearly demonstrate the possibility for arcing phenomena to occur in metal–solvent systems exposed to microwave irradiation.

In addition to liquid-phase synthetic organic chemistry applications, arcing phenomena in microwave chemistry play an important role in material sciences and nanomaterial research. For example, such effects involving highly conductive metals or metal oxides have been implicated in the formation of core/shell metal/carbon nanoparticles,^12^, ^13^ Fe_3_O_4_/carbon composite nanomaterials,^14^ ZrB_2_ or metal oxide nanostructures,^15^, ^16^ carbon nanotubes,^17^ and metal fiber/polymer composite materials.^18^ Using carbon or graphite-type materials, which are both prone to exhibit electric discharges under microwave conditions,^19^ applications range from high-temperature pyrolysis reactions^20^ to the high-speed formation of graphene sheets^21^ and carbon nanoscrolls.^22^ Arcing phenomena may also be involved in microwave-assisted electrochemistry applications.^23^

Surprisingly, in the context of microwave-assisted synthesis, arcing phenomena in metal–organic solvent systems have not been investigated in detail, with the exception of the classical treatise by Whittaker and Mingos published in 2000.^8^ In this study, an open-vessel, multi-mode microwave system was used to investigate the effects that cause electric arcing, employing different amounts of Al powder in a variety of organic solvents. Our recent experience with microwave-induced arcing phenomena involving Mg metal and THF as the solvent,^12^ has prompted us to investigate these effects in more detail. Herein, we describe our investigations into electric discharge phenomena utilizing a high field density, sealed-vessel, single-mode microwave reactor with a built-in camera. Particular emphasis is placed on studying the discharges exhibited by different metals (Mg, Zn, Cu, Fe, Ni) of varying particle sizes and morphologies at different electric field strengths.

## Results and Discussion

### Electric discharges and microwaves

Arcing phenomena in metal–solvent systems under microwave irradiation are critically dependent on many, in part difficult to control and ill-defined, factors, including, for example, number, size, morphology and surface conditions of the metal particles, ionization energy, purity and dielectric loss tangent of the solvent, and, of course, the applied magnetron output power.^8^ The external electric field causes an electric current in the conducting material and positive and negative charges accumulate on opposite sides of the material, creating a field opposing the applied electric field.^7^, ^24^, ^25^ In electrostatic equilibrium, the electric field created by the charges inside the material and the external electric field are equal, and the net electric field is zero everywhere inside the conductor (due to the finite conductivity of nonperfect conductors, the electromagnetic field does not vanish immediately but decays exponentially inside the material: penetration depth).^7^ The charges on the conductor move entirely to the conductor's surface, but the charge on the material′s surface does not distribute itself uniformly but rather the distribution depends on the detailed shape of the material. At sharp edges and submicroscopic irregularities, the surface charge density and thus the external electric field, may reach very high values, and an electrical breakdown in the medium around such sharp edges or points may occur, producing an electric spark or an electric arc. The maximum electric field strength that can be tolerated by the material surrounding the conductor is determined by its dielectric strength. The dielectric strength, however, is not a specific property of the dielectric but depends on several factors, such as temperature, frequency of the electric field—breakdown occurs more easily at low frequencies, because the fields have more time to accelerate background electrons—or the size, shape, material and surface conditions of the capacitor.^7^, ^24^, ^25^

In gases, breakdown occurs basically by collisional ionization.^26^ The equilibrium concentration of charged particles in gases at normal pressure is exceedingly low (≍10^3^ cm^−3^), and hence the electrical conductivity is very small (in the order of 10^−16^ to 10^−15^ S cm^−1^).^27^ However, in a strong electric field, these particles acquire kinetic energy large enough to ionize gas molecules. The newly charged particles ionize more molecules in an avalanche process, which can culminate in corona discharges, streamers, leaders, or in a spark or continuous arc that completely bridges the gap between differently charged particles.^26^

The dielectric strength of air in a uniform field is around 3 MV m^−1^.^27^ The dielectric strength of liquids can be orders of magnitudes higher than that of gases. Highly purified liquids have dielectric strengths as high as 100 MV m^−1^. For example, the dielectric strength of benzene is 163 MV m^−1^.^27^ The understanding of the electrical breakdown processes in liquids is not as advanced as for gases.^26^ According to one model, the mechanisms in pure liquids are supposed to be similar to those in gases (i.e., ionization discharge). A second, widely utilized model assumes the electric discharge in a liquid is a discharge in gas cavities, which are either already present in the liquid and on the electrodes, or newly formed under voltage exposure (e.g., by electrolysis, boiling, cavitation, and decomposition under electron bombardment). The mechanisms, however, depend on many factors (e.g., shape and material of the electrode) and can be significantly altered in the presence of solid and liquid impurities or dissolved gases.^26^

Furthermore, metal particles can be heated in a microwave field when present in a sufficiently small size.^7^ As described above, the free moving electrons in a conductor shield the electric field of microwaves, since microwave frequencies are far below the plasma frequency of metals and so microwaves are reflected, and the microwave interaction is restricted to the surface of the metallic sample only. The penetration depth of microwaves in metals is relatively small and typically varies from 0.1 to 10 μm (Table 1). However, metal particles with dimensions comparable to the penetration depth can couple with microwaves effectively and, therefore, can be heated very quickly in the microwave field.^28^

**Table 1:** Properties of Mg, Zn, Cu, Fe and Ni of relevance to microwave-induced arcing.^[a]^

	Mg	Fe	Ni	Cu	Zn
Molecular weight [g mol^−1^]	24.3	55.8	58.7	63.5	65.4
Density [g cm^−3^]	1.74	7.87	8.91	8.94	7.14
m.p. [°C]	650	1538	1455	1085	420
Penetration depth at 2.45 GHz [μm]^[b]^	2.2	3.2^[c]^	2.7^[c]^	1.3	2.5
Electric resistivity (ρ) at 20 °C [nΩ⋅m]	43.9	96.1	69.3	16.8	59.0
1 ^st^ ionization energy [kJ mol^−1^]	737.7	762.5	737.1	745.5	906.4
Work function (Φ) [eV]	3.66	4.67–4.81	5.04–5.35	4.48–4.94	3.63
Molar magnetic susceptibility (10^−6^) [cm^3^ mol^−1^]	+13.1	Ferro.	Ferro.	−5.46	−9.15
Pauling electronegativity	1.31	1.83	1.91	1.90	1.65

[a] Data taken from Ref. 7, 27. [b] Penetration depths were estimated with *d*=(*ρ*⋅(π⋅*f*⋅*μ_0_*⋅*μ′*)^−1^)^1/2^, where *ρ* is the electric resistivity, *f* is the frequency of the alternating field, and *μ_0_* is the permeability of free space (4⋅π⋅10^−7^ H m^−1^). The relative permeability (*μ′*) is assumed to be approximately one. For details, see Ref. 7. [c] The relative permeability (*μ′*) of ferromagnetic materials can be significantly greater than one, leading to a correspondingly lower penetration depth.

### Experimental design and microwave reactor

**Figure 1:**
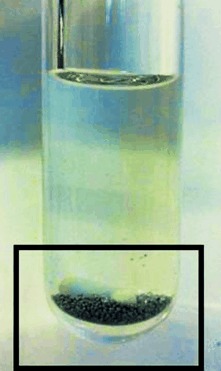
Image of a 10 mL cylindrical Pyrex® microwave vial containing Mg metal (120 mg), benzene (3 mL) and a magnetic stir bar. The black box indicates the area imaged by the built-in camera.

All microwave irradiation/arcing experiments in our studies were performed using a Monowave 300 single-mode microwave reactor from Anton Paar GmbH (Graz, Austria).^29^, ^30^ The instrument uses a maximum of 850 W magnetron output power at 2.45 GHz frequency and can be operated at a reaction temperature of 300 °C and a pressure of 30 bar. The high nominal magnetron power and the specific design of the single-mode cavity in this instrument generate significantly higher electric field strengths than other commercial single-mode microwave reactors.^10^, ^12^ The reaction temperature is monitored by an external infrared (IR) sensor housed in the side walls of the microwave cavity measuring the surface temperature of the reaction vessel. In addition, the temperature can be recorded by an internal fiber optic temperature probe (ruby thermometer) protected by a quartz immersion well inserted directly in the reaction mixture. In most cases, only the external IR sensor was used for temperature monitoring in order to avoid damage to the internal ruby thermometer as a result of arcing. Experiments were performed with a prototype instrument fitted with a camera in order to observe arcing phenomena under sealed-vessel conditions,^12^ and were carried out in constant power mode using a 10 mL Pyrex® vessel with magnetic stirring (Figure 1).

The microwave reactor employed can be operated either in temperature control or constant power mode. Using the temperature control mode, the reaction mixture is heated with the appropriate magnetron power to reach the target temperature either as fast as possible (AFAP mode) or within a certain time (ramp mode). Since the applied microwave power was expected to be a decisive factor in the discharge process, the experiments described herein were performed at constant magnetron power (typically for 30 s irradiation time), rather than applying a set temperature. Mg was chosen as a reference metal for our studies, because of its exceptional importance in organic synthesis. For further experiments, the diamagnetic metals Cu and Zn and the ferromagnetic metals Fe and Ni were selected (Table 1). The commercially obtained metals were separated into different size ranges with laboratory sieves (see Supporting Information for details). Additional experiments were performed with semiconducting materials, such as SiC and graphite.

Most of the arcing experiments were performed using benzene as the solvent (3 mL) since it is virtually microwave transparent and chemically sufficiently resistant.^31^ The microwave field mainly interacts with the metal particles dispersed in the solvent, and the bulk temperature, therefore, does not significantly rise above the boiling point during a 30 s microwave irradiation. As a result, the vapor pressure remains well within the pressure limit of the instrument (30 bar), although there are cases where, due to extensive solvent decomposition, the pressure inside the vial can increase significantly (see below). Additional solvents compatible with the metals used here and solvent-free conditions were also tested to study the influence of the dielectric media surrounding the metal on the breakdown process (Table 2).

**Table 2:** Properties of selected organic solvents of relevance to microwave-induced arcing.^[a]^

Solvent	b.p. [°C]	tan *δ*^[b]^	IE^[c]^ [eV]	Viscosity^[d]^ [mPa s]	Density^[e]^ [g mL^−1^]
Benzene	80	–	9.24	0.604	0.879
1,4-Dioxane	102	–	9.19	1.177	1.036
*n*-Hexane	69	0.02	10.13	0.300	0.659
Toluene	111	0.04	8.83	0.560	0.867
THF	66	0.047	9.38	0.456	0.889
EtOAc	77	0.059	10.01	0.423	0.901
MeCN	82	0.062	12.20	0.369	0.783
NMP	203	0.275	≤9.17	1.66	1.028
1-Butanol	117	0.571	9.99	2.544	0.811
MeOH	65	0.659	10.85	0.544	0.793
Ethylene glycol	197	1.351	10.16	16.1	1.113

[a] Data taken from Ref. 27. [b] Electric loss tan *δ* (25 °C, 2.45 GHz). [c] Ionization energy (IE). [d] Viscosity determined at 25 °C. [e] Density measured at 20 °C.

In general, bulk metals do not couple directly with microwave energy but readily reflect the incident energy.^7^ Accordingly, no arcing was observed when the metal–solvent mixture was not stirred and the metal particles remained in electrical contact at the bottom of the vial. In most cases, a good dispersion of the metal particles was achieved by applying a stirring speed of 600 rpm, and this setting was used for all experiments described below.

To eliminate the risk of an ignition of the metal–solvent or solvent decomposition products (e.g., the autoignition temperature of Mg ribbon is approximately 630 °C), the metal–solvent mixture was thoroughly degassed with Ar or N_2_ to remove any O_2_.

In a uniform field, the onset of ionization usually immediately leads to a complete breakdown of the dielectric medium.^25^ In the microwave field, however, various manifestations of audible and visible discharges were apparent long before a complete breakdown of the medium occurred. Depending on the factors described below, local electric discharges (i.e., corona discharges) or extended electrical breakdowns (i.e., arcs) were observed. A local ionization of the solvent surrounding the conductor created plasma as a uniform white–blue sheath around the metallic particle. The fast reversal of the field, though, precluded a complete breakdown of the whole gap to an arc—the electron drift velocity in an avalanche is in the order of 10^7^ cm s^−1^, and the total time necessary for the development of avalanches, avalanche–streamer transitions, and streamer propagations, between electrodes of a distance of 1 cm, for example, is about 100 ns, hence, much larger than the period of a 2.45 GHz microwave.^25^ The lifetime of the individual coronas was shorter than the exposure time of the installed digital camera (15–30 frames per second).^32^ Under harsher conditions, however, violent and clearly audible sparks and arcs were observed. Arcs were often accompanied by a visible change of the morphology and surface appearance of the metal and considerable decomposition/carbonization of the solvent. Under these conditions, the pressure in the sealed microwave vial increased rapidly during the arcing periods as a result of the formation of low-molecular-weight solvent decomposition products.^12^ The appearance of the discharges, such as color, brightness and size, differed somewhat for different metals (see below). However, for ease of reference and for a qualitative comparison of the intensity of the discharges observed in the individual experiments, the discharge phenomena were classified into ten discharge levels (Table 3 and Figure S1, Supporting Information).

**Table 3:** Classification of the observed discharges into ten different levels.^[a]^

Discharge level	Description
0	no discharges.
1	1–10 discharges s^−1^; mainly corona discharges.
2	10–30 discharges s^−1^; mainly corona discharges.
3	30–60 discharges s^−1^; mainly corona discharges.
4	60+ discharges s^−1^; mainly corona discharges.
5	60+ discharges s^−1^; corona discharges and occasional arcs.
6	60+ discharges s^−1^; corona discharges and arcs, formation of small amounts of carbonaceous material.
7	violent arcing; formation of carbonaceous material and coloration of the solvent.
8	very violent arcing; formation of carbonaceous material and coloration of the solvent; pressure remained ≤10 bar.
9	extremely violent arcing; formation of tree-like structures composed of carbonaceous material; metal particles welded to one another and damage to the reaction vessel caused by metal welded onto the glass of the vessel is seen; pressure quickly increased to pressures ≥10 bar and experiments often had to be aborted before the set time of 30 s had elapsed.

[a] Experiments were performed in a prototype single-mode microwave reactor fitted with a built-in camera (30 s irradiation time). The discharge levels were assessed with the aid of movies recorded by the camera. Still images were used to count discharges. Vigorous discharges (discharge levels>7) were dominated by repetitive bright flashes throughout the vial. Pressure build-up and extent of solvent decomposition were taken into account in these cases.

### Magnesium–benzene systems

Based on our previous experience with electric discharges in a Mg–THF system,^12^ our initial experiments were performed using Mg particles (120 mg) in benzene (3 mL). Benzene, being completely microwave transparent (Table 2), was chosen to enable an exclusive interaction of the microwave field with the Mg metal and not with the solvent.^31^ The Mg particles were in the size range of 400–500 μm and were obtained by applying appropriate sieves (Table S1, Supporting Information). The benzene solutions were purged with Ar, and the vials were then immediately irradiated at constant 300 W for a period of 30 s. Depending on the factors described below, clearly audible discharges were observed, often accompanied by a visible change of the morphology and surface appearance of the metal. Therefore, metal and solvent were replaced after each experiment.

### Effect of magnetron power

**Figure 2:**
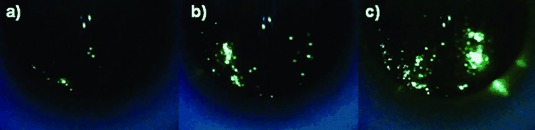
Effect of increasing microwave power on electric discharges: a) 200 W, b) 400 W, c) 600 W. Irradiation experiments were performed with Mg particles (120 mg) of 400–500 μm diameter in benzene (3 mL) for 30 s. For additional images and a correlation with the discharge levels described in Table 3, see Figures S2 and S3 in the Supporting Information.

Since the electric field strength is directly related to the administered magnetron power, discharges became, not surprisingly, more frequent and dramatic with increasing microwave power (discharge levels 1–4, Table 3). At low magnetron power (≍100 W), occasional audible and bright corona discharges were observed as uniform, concentric white–blue circles around the Mg particles (120 mg), indicating partial breakdown and ionization of the media surrounding the particle. When the power was increased to >100 W, the corona discharges became more frequent and increased both in area and intensity (Figure 2 a and 2 b). Ultimately, at higher field strengths (microwave power >500 W), complete electrical breakdown occurred and sparks or arcs covering an increasingly broad area were formed (Figure 2 c).

### Effect of number of particles

**Figure 3:**
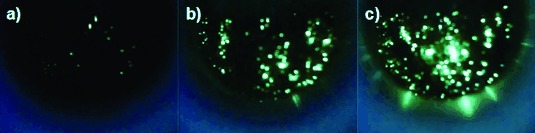
Effect of increasing amounts of Mg metal on electric discharges: a) 120 mg, b) 240 mg, c) 360 mg. Irradiation experiments were performed at 300 W constant power with 400–500 μm particles in benzene (3 mL) for 30 s. For additional images and a correlation with the discharge levels described in Table 3, see Figures S4 and S5 in the Supporting Information.

In the second phase of our experiments, the amount of Mg metal and, therefore, the number of individual particles were varied. Thus, the electric discharges were evaluated at 300 W constant power for varying amounts of Mg metal (60, 120, 240, 360, and 480 mg, 400–500 μm particle size) suspended in benzene (3 mL). As expected, the number of corona discharges increased with the number of metal particles (Figure 3 a–c; discharge levels 1–5, Table 3). Since the average distance between particles decreases with increasing particle number, occasional arcs spanning across two or more Mg particles occurred already at relatively low applied microwave power. Using constant magnetron power (300 W), however, the degree of arcing leveled off for very large amounts of Mg particles (>300 mg). This can be explained, at least in part, by the increasing difficulty to suspend additional Mg particles uniformly in the solvent. A significant amount of Mg particles accumulated at the bottom of the vial, more or less unaffected by the magnetic stirrer. The individual Mg particles remain in physical and electrical contact with each other at the bottom of the vial, and no observable discharges occurred. As mentioned above, when the solution was not stirred at all, no discharges were observed independent of the applied microwave power or number of Mg particles.

### Effect of Mg particle size and morphology

**Figure 4:**
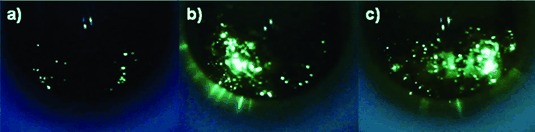
Effect of Mg particle size on electric discharges: a) 250–400 μm, b) 500–630 μm, c) 630–800 μm. Irradiation experiments were performed at 300 W constant power with Mg (120 mg) in benzene (3 mL) for 30 s. For additional images, see Figure S6 in the Supporting Information.

In the third phase of our investigations, the effect of particle size on the electric discharge phenomena was investigated. For these experiments, commercial Mg powder (40–800 μm) was sieved into seven particle-size ranges (Table S1, Supporting Information). Using a constant amount of Mg (120 mg), discharges became more pronounced with increasing size of Mg particles (discharge levels 1–5, Table 3), even though the number of particles decreases proportional to the third power of the size. With large particles (>500 μm), corona discharges were frequently accompanied by extended electrical breakdowns (Figure 4 b and 4 c). Mg particles <90 μm (powder) were no longer able to produce discharges, even when the full microwave power of 850 W was applied or the number of particles was increased.

**Figure 5:**
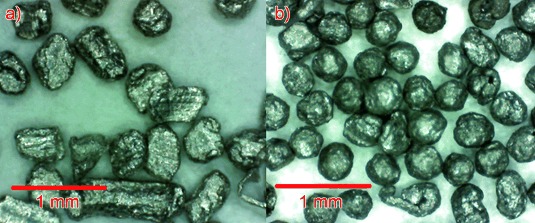
Images of Mg particles of comparable size but different morphology: a) Mg granules (particle size: 400–500 μm), b) spherical Mg grit (particle size: >355 μm). In contrast to the Mg granules, Mg grit does not generate discharges. See Tables S1 and S2 in the Supporting Information for details.

In addition to particle size, the morphology of the particles also appeared to be a decisive factor for the discharge process. As described above, the surface-charge density accumulates at sharp edges and points until the electric field around the edges exceeds the dielectric strength of the surrounding media. Ionization processes then occur and the surrounding dielectric media abruptly becomes conductive. In this context, it is interesting to note that sieved Mg particles (120 mg) of nearly spherical geometry (powder) did not produce visible discharges, in contrast to sieved samples of Mg granules of similar size and otherwise identical experimental conditions (Figure 5 a and 5 b). However, surface conditions such as surface conductivity, surface roughness and corrosion are also likely to have a significant effect on the discharge process.

It should be noted that our recent work on arcing phenomena in Mg–THF mixtures involved very large Mg granules of 2–3 mm particle size.^12^ In contrast to the experiments described above involving comparatively small particles, intense arcing accompanied by solvent carbonization was observed under those conditions (discharge levels 7–9, Table 3).

### Other metal–benzene systems

**Figure 6:**
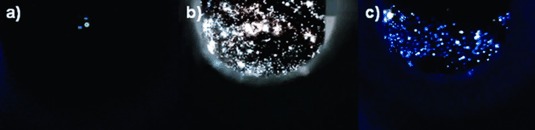
Effect of metal type on electric discharges: a) 120 mg Mg (discharge level 1, Table 3), b) 618 mg Cu (discharge level 7, Table 3), c) 493 mg Zn powder (discharge level 6–7, Table 3). Irradiation experiments were performed at 300 W constant power with 125–180 μm particle size in benzene (3 mL) for 30 s. The same number of particles (≍23 000–67 000) was used in all three cases. For additional images, see Figures S7 and S9 in the Supporting Information.

In comparison to the arcing observed with Mg metal, a similar behavior was also observed for diamagnetic metals such as Zn and Cu (Table 1). Discharges required a minimum particle size (≍40 μm), and the discharges became more violent with increasing number and size of particles (Tables S3–S6 and Figure S7 and S9, Supporting Information). However, when the particle size became larger than ∼100 μm, Zn and Cu invariably produced strong electric discharges independent of the particle size (discharge levels >6–7 at 300 W, Table 3). Due to the significantly higher densities of these metals (Table 1), the amounts of metal particles were adjusted accordingly, so that the number of particles remained roughly the same for all experiments, for example, for particles in the size range of 125–180 μm, ∼23 000–67 000 particles were added. Violent, bright white discharges, in particular for Cu (Figure 6 b), and white–purple discharges in the case of Zn (Figure 6 c), which frequently covered most of the reaction vial, appeared at constant magnetron powers as low as 300 W using benzene as the solvent and particles in the size range 125–180 μm (Figure 6). An important factor again was the morphology of the particles. For example, Zn particles in the form of needles exhibited considerably stronger discharges (discharge level 9, Table 3) compared to Zn particles of more spherical shape (discharge levels 6–7, Table 3) in the same size range at constant 300 W (Figure S7 and S8, Supporting Information).

In general, it can be expected that the material of the conductor in the microwave field plays a very important role in the discharge process by supplying electrons for initiating, sustaining, and completing the discharge.^7^, ^24^, ^25^ Under normal conditions, electrons are prevented from leaving the bulk material by electrostatic forces between the electrons and ions in the lattice, but there are several processes in which the required energy can be supplied to release the electrons (field emission, thermionic emission, electron emission by impact of ions or excited atoms, photoelectric emission).25a The energy required to remove an electron from a Fermi level is known as the work function (Table 1). However, the discharge levels found for different metals under comparable conditions (same size range and number of particles, and same nominal magnetron power) did not reveal any correlation with the work function of the metal (e.g., discharges were more dramatic with Cu (Φ=4.84–4.94 eV) than with Mg (Φ=3.66 eV)). Discharge processes are strongly influenced by both the liquid–electrode interface and bulk properties that include a variety of electric field- and temperature-dependent processes.^33^ As described above, surface conditions, such as surface regularity and cleanness, are important aspects, but a number of other factors (e.g., surface temperature, penetration depth, magnetic properties) likely have an influence on the occurrence of arcing for individual metals.

**Figure 7:**
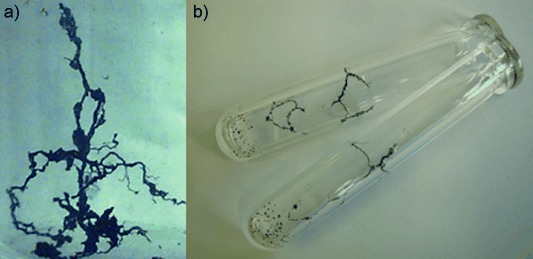
a) An electrical tree formed by Zn (300 W constant power, 493 mg, particle size: 180–250 μm, 3 mL benzene). b) “Lichtenberg figures” incorporated into a 10 mL Pyrex® microwave vial. The vial was cut lengthwise into two halves.

As Zn has a rather low melting point (420 °C), the violent electric discharges produced with the Zn needles in benzene (discharge level 9, Table 3) apparently melted the metal to some extent. At nominal power levels of 300 W and with particles in the size range of ∼150–250 μm, branched, tree-like structures (electrical trees or Lichtenberg figures) apparently made of metal and carbonized solvent were formed,^12^ which partly melted onto the Pyrex glass of the reaction vessel, for which the softening point lies around 820 °C (Figure 7). On occasion, electrical trees were also observed with large Mg turnings (2–3 mm particle size) and even with metals that exhibit high melting points, such as Fe (1538 °C) and Ni (1455 °C) (see below). This confirms the extreme local temperatures occurring in these electric discharge experiments.^8^ The overall heating rate, however, was virtually unaffected by the degree of arcing, and the bulk temperature of the solvent remained rather moderate (<150 °C) during experiments with 30 s microwave irradiation time at 300 W. The violent discharges were accompanied by considerable decomposition/carbonization of the surrounding solvent as previously described (discharge level 9, Table 3).^8^, ^12^

The ferromagnetic metals Fe and Ni (Table 1) had a tendency to stick to the magnetic stir bar and, thus, a proper dispersion in the solvent could not be achieved. When the particles remained in electrical contact to each other on the stir bar, discharges did not occur. However, as soon as some particles were released from the stir bar, for example, when vapor bubbles formed around the stirrer at the boiling point of the solvent, very violent arcing immediately started (discharge levels 9, Table 3). The discharges were accompanied by considerable solvent decomposition at constant 300 W, irrespective of the particle sizes (>40 μm). The discharges were dominated by complete dielectric breakdown of the benzene solvent with frequent formation of sparks extending virtually through the entire volume of the vial (Figures S10 and S11, Supporting Information).

**Figure 8:**
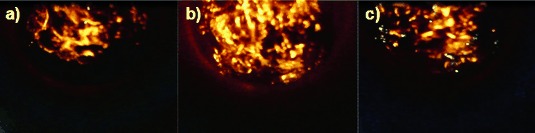
Very small ferromagnetic metal particles appear to glow in the microwave field (300 W constant power, particle size <40 μm, 3 mL benzene): a) 615 mg Ni, b) 542 mg Fe. c) Discharges were occasionally observed between the red glowing spots, shown here in the case of Fe (542 mg).

In addition to the effects of the electric field component of microwave irradiation, the impact of the associated magnetic field component has to be taken into account when magnetic materials are exposed to microwaves.^7^, ^34^ Here, the corresponding terms are the permeability (*μ′*) and the magnetic loss factor (*μ′′*), which represents the magnetic loss due to relaxation and resonance processes under the influence of an alternating magnetic field. The effects of the magnetic field component may be of significant importance for the discharge phenomena observed with ferromagnetic Fe or Ni.^7^ Orange–red glowing spots between the bright white or white–blue discharges were occasionally observed for small Fe and Ni particles. These spots became more prominent when the particle size decreased and were particularly intense for very small Fe and Ni powder (<40 μm; Figure 8 a and 8 b and Table S7, Supporting Information). Apparently, these small particles were heated extremely rapidly in the microwave field to very high temperatures at a magnetron power of 300 W (ferromagnetic materials are heated in particular by the magnetic field component)^7^ and started to glow virtually immediately after the microwave power was turned on (Figure S13, Supporting Information). For example, spherical Fe particles of 5 μm diameter would have a heat capacity of ∼0.23×10^−9^ J K^−1^ at room temperature and, thus, can be expected to be heated very rapidly in a high density microwave field. The orange–red glowing spots were interspersed by occasional bright blue local plasma discharges (Figure 8 c). This phenomenon was observed only during the first ∼5 s of microwave irradiation. Thereafter, bright white flashes throughout the entire vial, similar to those observed for larger particles, superseded the orange glowing spots.

Branched structures similar to the one shown in Figure 7 a were often formed in experiments with Fe and Ni particles of all tested size ranges (<355 μm) at 300 W magnetron power, and the benzene solution frequently turned orange. Although the bulk temperature, as determined by the IR sensor, did not exceed the boiling point of the solvent considerably during microwave irradiation (120–150 °C), the experiments often had to be aborted after a few seconds since the pressure increased sharply (>20 bar were reached within 10–25 s). Considerable pressure remained inside the vial after cooling the mixture back to room temperature (3–5 bar), indicating the formation of volatile solvent decomposition products.^12^ GC–MS analysis revealed the formation of small amounts of a variety of aromatic and polyaromatic compounds, in particular biphenyl, naphthalene and phenylacetylene.

In an additional series of experiments, the arcing behaviors of other strongly microwave-absorbing materials were briefly evaluated. Violent electrical discharges in benzene were observed with granules (1500–2500 μm) of silicon carbide (SiC), a semi-conducting ceramic material that has become increasingly popular for a variety of applications in microwave chemistry,^35^ at nominal microwave powers >100 W (Figure S12, Supporting Information). SiC is strongly microwave absorbing and can be utilized at extremely high temperatures due to its high melting point (2700 °C) and chemical inertness. Several studies have described the use of SiC as so-called passive heating elements or susceptors in microwave chemistry.^36^ These discharges were again accompanied by the formation of significant amounts of black carbonaceous material.

No arcing was observed with SiC powder or samples of graphite (20 μm), however, under these conditions, a very little arcing was indeed observed for Pd/C (20 μm) catalysts (discharge level 1, Table 3). The fact that discharges were indeed observed for Pd/C could have significant consequences for a variety of important microwave-assisted synthetic transformations that are catalyzed by this very common transition-metal catalyst, including hydrogenations and carbon–carbon cross-coupling chemistry.^37^ In fact, strong electric discharges were recently reported during a microwave-assisted Suzuki–Miyaura coupling reaction catalyzed by Pd/C in toluene as the solvent.^38^

### Copper–organic solvent systems

Selecting Cu as a comparatively unreactive metal, we next examined the occurrence of microwave-induced discharges in different organic solvents. For these experiments, the size and number of particles were kept constant (180–250 μm, 618 mg) in order to exclusively investigate the effect of the solvent on the arcing process. The power required for the onset of arcing depended mainly on the loss tangent (tan δ) of the solvent (Table 2),^31^ with significantly more arcing observed for microwave-transparent or low-absorbing solvents using otherwise identical conditions (an exception was dioxane). The loss tangent of a dielectric material quantifies its inherent dissipation of electromagnetic energy. Solvents with high loss tangents absorb microwaves very efficiently.^5^, ^31^ The microwave power is mainly dissipated in the solvent, and the electric field is rapidly attenuated on passing through the mixture. For example, with microwave power of less than 100 W, no discharges were observed in highly microwave-absorbing solvents, such as *N*-methyl-2-pyrrolidone (NMP), ethylene glycol or methanol. However, when the microwave power was increased to higher levels (>100 W), discharges did occur and often became quite dramatic (Table 4).

**Table 4:** Discharges in different solvents with copper particles (618 mg, 180–250 μm) at different power levels.

Solvent^[a]^	MP^[b]^ [W]	DL^[b]^	Solvent^[a]^	MP^[b]^ [W]	DL^[c]^
Benzene	100	2	MeCN	100	1–3
	200	5		200	5–7
	300	8		300	6–7
1,4-Dioxane	100	0	NMP	100	0
	200	1–2		200	2–6
	300	3–4		300	6–7
*n*-Hexane	100	1–5	1-Butanol	100	1
	200	2–5		200	5–6
	300	5–6		300	5–6
Toluene	100	1–5	MeOH	100	0
	200	1–5		200	0
	300	4–5		300	4–5
THF	100	3–4	Ethylene glycol	100	0
	200	4–5		200	0
	300	4–7		300	1–5
EtOAc	100	1–3	–	–	–
	200	5–6	–	–	–
	300	5–6	–	–	–

[a] For solvent properties (b.p., tan *δ*), see Table 2.

[b] Microwave power (MP).

[c] Discharge level (DL). For definitions, see Table 3.

For solvents with comparable loss tangents and at low levels of applied microwave power (≍100 W), there was a loose correlation between the severity of discharges and the boiling point of the solvent. The discharges were generally more pronounced in the lower boiling solvents and became more dramatic as the temperature of the solvent in the closed vessel increased. Whittaker and Mingos demonstrated a broadly linear relationship between the discharge level and the boiling point for different classes of solvents with low loss tangents, such as straight chain alkanes, cycloalkanes and aromatic solvents.^8^ They hypothesized that the electric discharges actually occur through the solvent vapor. The metal surface can be heated in the microwave field very rapidly (heating rates as high as 100 °C s^−1^ were reported),^7^, ^39^ and vapor bubbles are formed at nucleation sites of the hot surface. The dielectric constant of the formed bubbles is considerably lower than that of the bulk solvent, and the electric field across the bubbles is correspondingly higher.^40^ Moreover, the dielectric strength is significantly lower in the vapor bubbles than in the surrounding liquid phase. When the field in the bubble becomes equal to the gaseous ionization field, discharge takes place and breakdown might follow. Thus, a correlation between boiling point and arcing level is expected. Local evaporation of the solvent on the hot metal surface can also occur in high boiling solvents, but bubbles released from the surface rapidly collapse in the cold surrounding liquid. At higher power levels (>100 W), arcing of variable degrees was produced in all tested solvents (Table 4). The discharges often led to decomposition of the solvent with formation of black carbonaceous materials and discoloration of the liquid phase. The pressure in the sealed microwave vial rapidly increased during these experiments, probably due to the formation of low-molecular-weight decomposition products.

Generally, many factors influence the arcing process in metal–liquid phase systems (breakdown strength and ionization potential of the solvent, loss tangent, purity, dissolved gasses, stirring characteristics, viscosity) and the breakdown strength and mechanisms strongly depend on the amount and nature of the impurities present.^26^ Therefore, these phenomena are extremely difficult to quantify and correlate.

### Discharge phenomena without solvent

**Figure 9:**
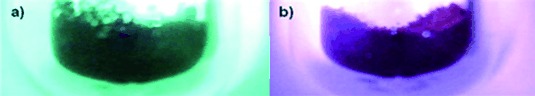
Discharges of Cu powder (618 mg, 50 W microwave power, 180–250 μm particle size) in different atmospheres: a) Ar, b) N_2_.

Microwave-induced arcing involving Cu metal was particularly violent when these experiments were carried out in the absence of solvent. Cu particles in the size range of 180–250 μm displayed dramatic, bright white and aquamarine blue discharges in an Ar environment upon irradiation at only 50 W microwave power (Figure 9 a). Neutral and ionized Ar excited in an electrical discharge has several blue emission lines in the spectrum, and blue is the typical color of an Ar plasma discharge tube.^41^ The breakdown strength of Ar is about 1/5 of the breakdown strength of air. In contrast, the dielectric strength of N_2_ is slightly above that of air.^27^ Cu irradiated at constant 50 W in a N_2_ atmosphere gave bright pink discharges across the entire cross-section of the vial (Figure 9 b). The discharges were, however, much less frequent than they were in an Ar atmosphere. Although the morphology of the Cu particles did not change appreciably after irradiation in N_2_, the characteristic red–brown color of the Cu metal changed to violet (150 W; IR temperature of 150 °C after 30 s) and grey (200 W; IR temperature of 190 °C after 30 s).

**Figure 10:**
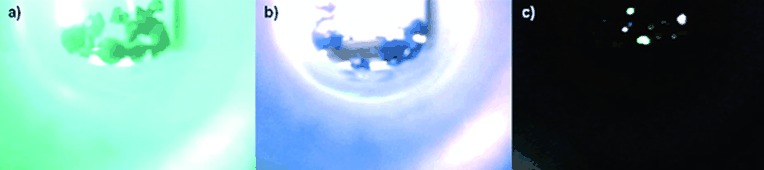
Discharges of SiC granules (221 mg, 1500–2500 μm particle size) in an Ar atmosphere at a) 50 W and b) 100 W, and c) N_2_ atmosphere at 50 W microwave power.

A very similar behavior to that of Cu was also observed with SiC (1500–2500 μm particle size) in N_2_ and Ar atmospheres. Extended aquamarine blue discharges were formed in an Ar environment at low power levels (50 W, Figure 10 a). At higher power levels (100 W) the discharges became more frequent and brighter, and the color turned to a lavender blue (Figure 10 b). In a N_2_ environment, on the other hand, faint yellow, local corona discharges were observed at magnetron powers of 50 and 100 W without the formation of sparks or arcs (Figure 10 c).

## Conclusion

In summary, we have presented a qualitative description of discharge phenomena in metal–solvent systems in a high field density, single-mode microwave reactor. Metal particles reflect microwaves and concentrate the field at sharp edges and points. When the field exceeds the dielectric strength of the surrounding solvent, breakdown processes occur. Various factors, such as amount, size, morphology and physical properties of the conductor or semiconductor influence the breakdown processes in a given solvent at a given magnetron output power.

The discharge phenomena for diamagnetic and paramagnetic materials strongly depended on the size of the used particles. With small particles, short-lived (i.e., life span of some fractions of a second) corona discharges were observed as bright uniform sheets around the metal, but did not lead to a complete breakdown of materials. Under these conditions, the period of a 2.45 GHz microwave is too short for the development of streamer and streamer propagation. Under high power conditions or with large particles and/or a large number of particles, however, complete breakdown was observed. The bright sparks and arcs were frequently accompanied by solvent decomposition and formation of considerable amounts of graphitized material. For ferromagnetic particles the magnetic field component plays a critical role. In particular, small Fe and Ni powders with particles size <40 μm were heated very rapidly and started to glow in the microwave field. This suggests local temperatures in excess of 500 °C.

There is considerable interest in electrical breakdown processes under microwave conditions for a variety of reasons. Several authors recently described liquid-phase applications of microwave-induced discharges or extreme local temperatures (i.e., hot spots), in particular in the material sciences and nanomaterial research.^11^–^23^ However, the influence of arcing phenomena in microwave chemistry may be more frequent than generally assumed. We suspect that in a number of published cases where zero-valent metals (or other highly electrically conductive materials) have been employed under microwave conditions, electrostatic discharges might play an important role.^1^–^3^ We have re-evaluated the recently reported microwave-assisted dehalogenation of 2-chloroethylbenzene, performed in the presence of metal and metal oxide powders such as Fe, Ni, and Fe_3_O_4_ in decaline as the solvent (Scheme S1, Supporting Information).^42^ Employing the single-mode microwave instrument with the built-in camera, in all these cases intense, strong arcing phenomena were observed under the published reaction conditions. These discharges could potentially be responsible for the proposed microwave effects seen in these dehalogenations (Figure S13, Supporting Information).^42^ Arcing phenomena could, therefore, also play a role in the microwave-assisted formation of inorganic metal or metal oxide nanomaterials, since some of these products are typically highly electrically conductive.^43^

## Experimental Section

**Materials**: Solvents were obtained from standard commercial vendors and used without any further purification. All solvents used in this study were dry solvents stored over molecular sieve. Metal particles were obtained from standard commercial vendors and were sieved with analytical sieves of international standard size (Supporting Information).

**Microwave irradiation/discharge experiments**: All microwave irradiation/discharge experiments were performed using a Monowave 300 single-mode microwave reactor from Anton Paar GmbH (Graz, Austria) as previously described.^29^, ^30^ The appropriate amount of sieved metal particles and solvent (3 mL) were added into an oven-dried 10 mL microwave Pyrex® vial equipped with a stirring bar . The vial was sealed with a PEEK snap cap and a polytetrafluoroethylene (PTFE)-coated silicon septum, and the suspension was purged with Ar or N_2_ for 5 min via a needle. The vial was subsequently irradiated at constant magnetron power with an irradiation time of 30 s, maximum temperature of 150 °C and stirrer speed of 600 rpm. The built-in camera^32^ was used to record the discharge phenomena, and the external IR sensor was employed for temperature monitoring in order not to damage the internal fiber-optic (ruby) thermometer as a result of arcing. After the reaction time had lapsed, the vial was cooled with compressed air and removed from the microwave cavity.

## Supporting Information

Detailed facts of importance to specialist readers are published as ”Supporting Information”. Such documents are peer-reviewed, but not copy-edited or typeset. They are made available as submitted by the authors.
