# Supplementary material for: Characterization of Microwave-Induced Electric Discharge Phenomena in Metal–Solvent Mixtures
Source: ChemistryOpen. 2012 Feb 10;1(1):39–48. doi: 10.1002/open.201100013 (PMC3922439; doi:10.1002/open.201100013)
Supplement: Supplementary file 1 [file open0001-0039-SD1.pdf]

ChemistryOPEN

Supporting Information

© Copyright Wiley-VCH Verlag GmbH & Co. KGaA, 69451 Weinheim, 2012

Characterization of Microwave-Induced Electric Discharge Phenomena in Metal–Solvent Mixtures

Wen Chen,[a, b] Bernhard Gutmann,[a] and C. Oliver Kappe*[a]

open_201100013_sm_miscellaneous_information.pdf

Table S1. Listing of sieved Mg particles[a]

Nr.	Supplier	Order Nr.	Commercial description Shape	Commercial description Size [µm]	<40	40-90	90-125	125-180	180-250	250-355	>355
1	Alfa Aesar	00869	powder	150-850						1.1[b]	1.2-1.4[b]
2	Alfa Aesar	36194	powder	75-150		2.1	2.2	2.3			
3	Aldrich	13112	powder	/	3.1	3.2	3.3	3.4			
4	Aldrich	254126	granule	63-850	4.1	4.2	4.3	4.4	4.5	4.6	
5	Aldrich	63040	grit	106-600				5.1	5.2	5.3	5.4
6	Alfa Aesar	010232	turning	3200							6[c]

[a] The commercial metal particles were sieved with analytical sieves of international standard size (ISO 3310-1). The studies described in Section C and shown in Figure 2-4 and Figure S1-S6, were performed with Mg powder from Alfa Aesar (Particle Nr. 1 and 2). [b] The particles were sieved with an additional set of sieves in the size ranges of 250-400, 400-500, 500-630 and 630-800 µm. [c] Mg turnings had an average particle size of around 2-3 mm. These particles were used in our previous work on Grignard reagent formation under microwave irradiation.[S1]

Table S2. Discharge levels for the tested Mg particles[a]

Entry	Amount [mg]	Power [W]	Size [µm]	Particle Nr.	Discharge level	Particle Nr.	discharge level
1	120	300	< 40	**-**	-	**4.1**	0
2	120	850					0
3	360	300					0
4	120	300	40-90	**2.1**	0	**4.2**	1--2
5			90-125	**2.2**	0--1	**4.3**	2
6			125-180	**2.3**	0--1	**4.4**	2--3
7			180-250	**-**	-	**4.5**	2--3
8			250-400	**1.1**	2--3	**4.6*[b]***	3
9			400-500	**1.2**	2--3	**-**	-
10			500-630	**1.3**	4--5	**-**	-
11			630-800	**1.4**	4--5	**-**	-
12	120	300	125-180	**3.4**	0		
13	120	850			0		
14	360	300			0		
15	120	300	> 355	**5.4**	0		
16	600	700			0		

[a] Irradiation experiments were performed for 30 s in 3 mL of benzene and under an Argon atmosphere. Mg granules (Particle Nr. 4, Table S1) gave discharge levels comparable to Mg powder (Particles Nr. 1 and 2, Table S1) in the same size range and under identical conditions (entry 1 to 11). Small Mg granules or powder (< 40 µm) did not give electrical discharges. In contrast, Mg powder (Particle Nr. 3) and grit (Particle Nr. 5) did not show any discharges under the tested conditions (Entry 12 to 14 and 15 to 16, respectively) indicating the importance of the morphology of the metal particles on the discharge process (Figure 5).[b] size range was 250-355 µm.

Table S3. Listing of sieved Zn particles[a]

Nr.	Supplier	Order Nr.	Commercial description Shape	Commercial description Size [µm]	<40	40-90	90-125	125-180	180-250	250-355	>355
1	Fisher Scientific	Z/0450/60	powder	/	1.1	1.2	1.3	1.4	1.5		
2	Aldrich	243477	granule	150-600				2.1	2.2	2.3	2.4
3	Strem Chemials	93-3060	powder	/	3.1	3.2					

[a] The commercial metal particles were sieved with analytical sieves of international standard size (ISO 3310-1).

Table S4. Discharge levels for the tested Zn particles[a]

Entry	Amount [mg]	Size [µm]	Particle Nr.	Discharge level	Max Temp. [°C][b]	Max Pres. [bar][c]
1	493	< 40	**1.1**	0	75	0
2	493	40-90	**1.2**	1	72	0
3	493	90-125	**1.3**	6--7	109	2
4	120	125-180	**1.4**	6--7	85	1
5	493			6--7	103	1
6	493	180-250	**1.5**	6--7	106	3
7	493	150-180	**2.1**	9	149	31
8	493	180-250	**2.2**	9	136	27
9	493	250-355	**2.3**	8	137	7
10	120	355-600	**2.4**	7	81	1
11	493			8	130	6
12	493	40-90	**3.2**	0--1	76	1

[a] Irradiation experiments were performed for 30 s at 300 W constant power in 3 mL of benzene under an Argon atmosphere. Similar to what was observed for Mg particles, small Zn particles (<40 µm, entry 1) did not give electrical discharges. With larger particles (>90 µm), however, violent discharges were observed. Small amounts of black carbonaceous powder where formed in these experiments and the pressure increased rapidly, presumably due to the formation of volatile solvent decomposition products. Very small amounts of organic decomposition products were occasionally detectable by GC-MS (in particular biphenyl, naphthalene and phenylacetylene). [b] Max. temperature during 30 s microwave irradiation. [c] Max. pressure during 30 s microwave irradiation.

Table S5. Listing of sieved Cu particles[a]

Nr.	Supplier	Order Nr.	Commercial description Shape	Commercial description Size [µm]	<40	40-90	90-125	125-180	180-250	250-355	>355
1	Alfa Aesar	00908	powder	150-425				1.1	1.2	1.3	1.4
2	Alfa Aesar	42623	powder	45-150	2.1	2.2	2.3				
3	Aldrich	207880	powder	< 75	3.1	3.2					

[a] The commercial metal particles were sieved with analytical sieves of international standard size (ISO 3310-1).

Table S6. Discharge levels for the tested Cu particles[a]

Entry	Amount [mg]	Size [µm]	Particle Nr.	Discharge level	Max Temp. [°C][b]	Max Pres. [bar][c]
1	619	< 40	**2.1**	0	70	0
2	619	40-90	**2.2**	4	118	4
3	619	90-125	**2.3**	5	112	2
4	120	125-180	**1.1**	3--5	85	0
5	619			7	120	5
6	619	180-250	**1.2**	8	122	5
7	619	250-355	**1.3**	8	109	3
8	120	> 355	**1.4**	1	63	0
9	619			8	103	2
10	619	< 40	**3.1**	0	71	0
11	619	40-90	**3.2**	0	71	0

[a] Irradiation experiments were performed for 30 s at 300 W constant power in 3 mL of benzene and under an Argon atmosphere. Small Cu particles (around 40 µm, entries 1, 10 and 11) did not give electrical discharges. Small amounts of black carbonaceous powder where formed with particles bigger than around 180 µm (entries 6, 7 and 9). [b] Max. temperature during 30 s microwave irradiation. [c] Max. pressure during 30 s microwave irradiation.

Table S7. Listing of sieved Fe particles[a]

Nr.	Supplier	Order Nr.	Commercial description: Shape	Commercial description: Size [µm]	<40	40-90	90-125	125-180	180-250	250-355
1	Alfa Aesar	14183	powder	< 850	1.1	1.2	1.3	1.4	1.5	1.6
2	Alfa Aesar	41830	sponge	150-300	2.1	2.2	2.3	2.4	2.5	2.6
3	Acros	197811000	powder	< 212	3.1	3.2	3.3	3.4	3.5	
4	Merck	3800	powder	-	4.1	4.2	4.3	4.4		
5	Aldrich	209309	powder	< 45	5.1	5.2				
6	Aldrich	C3518	powder	4.5-5.4	6					

[a] The commercial metal particles were sieved with analytical sieves of international standard size (ISO 3310-1).

Table S8. Discharge levels for the tested Fe particles[a]

Entry	Amount [mg]	Size [µm]	Particle Nr.	Discharge level	time [s]	Max Temp. [°C][b]	Max Pres. [bar][c]
1	542	< 40	1.1	9	25	150	26
2	542	40-90	1.2	9	26	164	28
3	542	90-125	1.3	4--5	30	154	6
4	120	125-180	1.4	0--1	30	97	1
5	542			4--5	30	135	5
6	542	180-250	1.5	0--1	30	112	2
7	542	250-355	1.6	0--1	30	109	2
8	542	< 40	2.1	9	11	113	14
9	542	40-90	2.2	9	9	106	11
10	542	90-125	2.3	9	10	95	11
11	120	125-180	2.4	0	30	86	0
12	542			9	21	136	16
13	542	180-250	2.5	9	14	126	13
14	542	250-355	2.6	9	26	122	15
15	542	< 40	3.1	9	21	137	15
16	542	40-90	3.2	8	30	150	7
17	542	90-125	3.3	9	23	139	14
18	120	125-180	3.4	0	30	88	0
19	542			9	10	90	10
20	542	180-250	3.5	9	9	90	11
21	542	< 40	4.1	9	24	149	20
22	542	40-90	4.2	5	30	157	9
23	542	90-125	4.3	5	30	142	6
24	120	125-180	4.4	0--1	30	117	1
25	542			4	30	138	5
26	542	< 40	5.1	9	18	139	20
27	542	40-90	5.2	9	26	155	21
28	542	4.5-5.4	6	7[d]	30	134	7

[a] Irradiation experiments were performed for the indicated time at 300 W constant power in 3 mL of benzene as solvent and under an Argon atmosphere. Fe particles tended to stick on the magnetic stir bar and thus a proper dispersion in the solvent could not be achieved. When the particles remained in electrical contact to each other on the stir bar discharges did not occur. But as soon as some particles were released from the stir bar, very violent arcing immediately started independent of the particle size. Variable amounts of black carbonaceous powder were formed and the pressure increased sharply, presumably due to the formation of volatile solvent decomposition products. Small amounts of organic decomposition products were occasionally detectable by GC-MS (in particular biphenyl, naphthalene and phenylacetylene). [b] Max. temperature during microwave irradiation. [c] Max. pressure during microwave irradiation. [d] 100 W constant power.

Table S9. Listing of sieved Ni particles[a]

Nr.	Supplier	Order Nr.	Commercial description: shape	Commercial description: size [µm]	<40	40-90	90-125	125-180	180-250	250-355	>355
1	Alfa Aesar	10579	powder	150-300		1.1	1.2	1.3	1.4		
2	Aldrich	203904	powder	< 150	2						
3	Aldrich	266981	powder	5	3						

[a] The commercial metal particles were sieved with analytical sieves of international standard size (ISO 3310-1).

Table S10. Discharge levels for the tested Ni particles[a]

Entry	Amount [mg]	Size [µm]	Particle Nr.	Discharge level	time [s]	Max Temp. [°C][b]	Max Pres. [bar][c]
1	615	40-90	1.1	9	21	141	22
2	615	90-125	1.2	9	15	137	23
3	120	125-180	1.3	0--1	30	109	2
4	615			9	23	148	30
5	615	180-250	1.4	5	30	133	5
6	615	<40	2	9	11	119	13
7	205	5	3	9[d]	20	145	15

[a] Irradiation experiments were performed for the indicated time at 300 W constant power in 3 mL benzene under an Argon atmosphere. Like the Fe particles, Ni particles tended to stick on the magnetic stir bar and no discharges occur. As soon as some particles were released from the stir bar very violent arcing started independent of the particle size. Variable amounts of black carbonaceous powder were formed and the pressure increased sharply, presumably due to the formation of volatile solvent decomposition products. Small amounts of organic decomposition products were occasionally detectable by GC-MS (in particular biphenyl, naphthalene and phenylacetylene). [b] Max. temperature during microwave irradiation. [c] Max. pressure during microwave irradiation. [d] 150 W constant power.

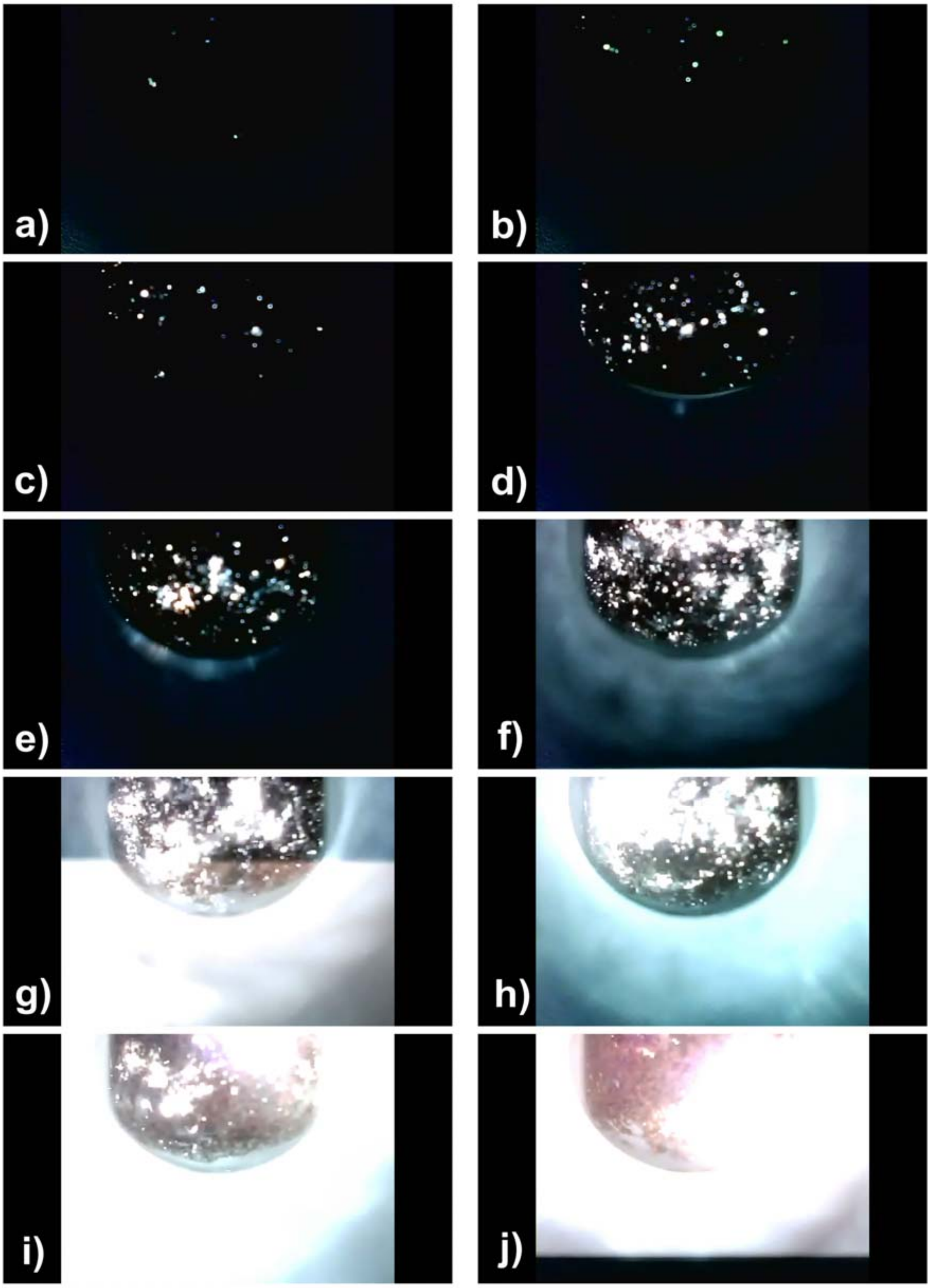

Figure S1. Images of discharges corresponding to discharge levels from 1 to 9: (a) to (f) discharge levels 1 to 6 using Cu (618 mg Cu particles **1.2**, 180-250 µm) in hexane (THF in case of (f)) at different MW power; (g) to (j) Representative images of violent arcing (discharge levels 7 to 9) with Cu (618 mg Cu particles **1.2**, 180-250 µm) in NMP. Extent of solvent decomposition and pressure build-up were taken into account in assessing these levels.

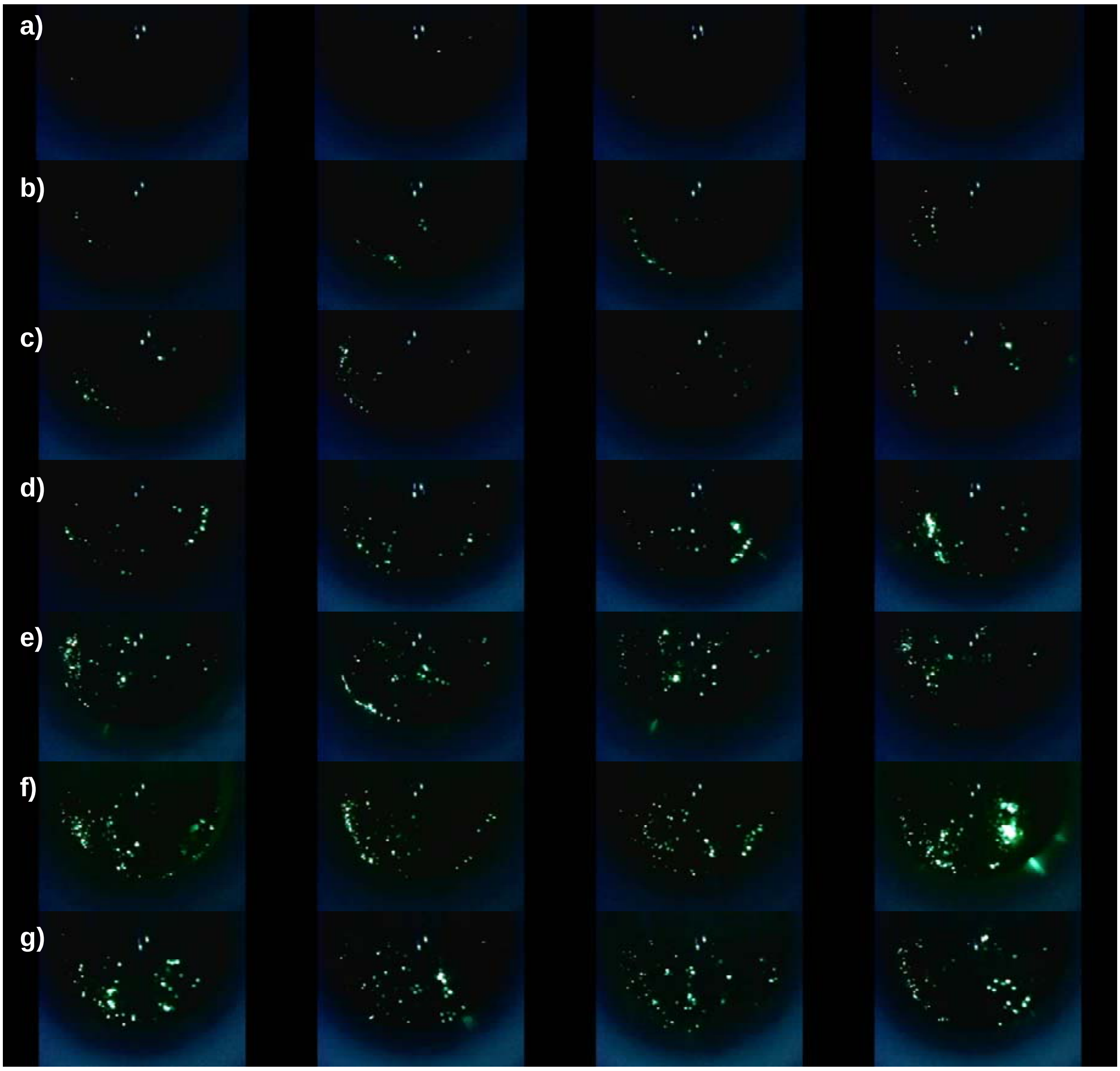

Figure S2. Effect of increasing microwave power (120 mg Mg particles, 400–500 µm, 3 mL benzene, particles 1.2 in Table S1) on electrical discharges. (a) 100 W, (b) 200 W, (c) 300 W, (d) 400 W, (e) 500 W, (f) 600 W, (g) 850 W. Irradiation experiments were performed for 30 s. Shown are still images of the videos obtained with the build-in camera.

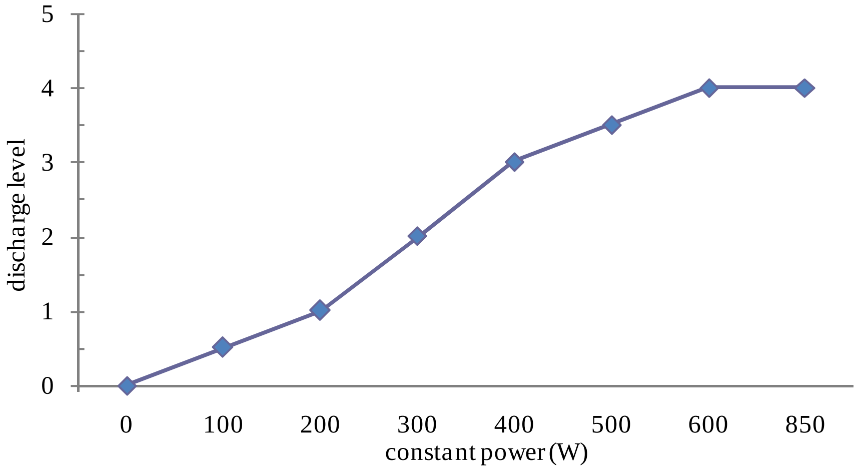

Figure S3. Correlation of microwave power with discharge level (Table 3). For experimental conditions, see Figure S2.

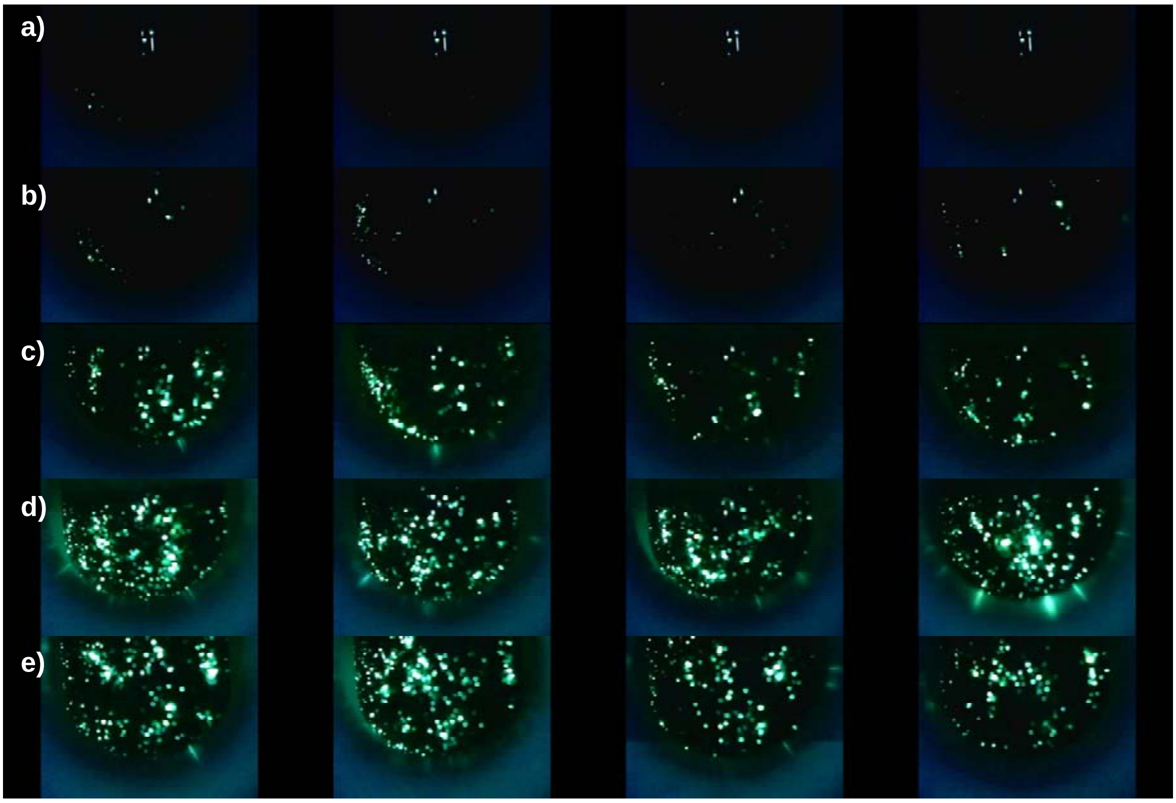

Figure S4. Effect of increasing the amount of Mg metal (300 W constant power, 400–500 µm, 3 mL benzene, Particles 1.2 in Table S1) on electrical discharges. (a) 60 mg, (b) 120 mg, (c) 240 mg, (d) 360 mg, (e) 480 mg. Irradiation experiments were performed for 30 s. Shown are still images of the videos obtained with the build-in camera.

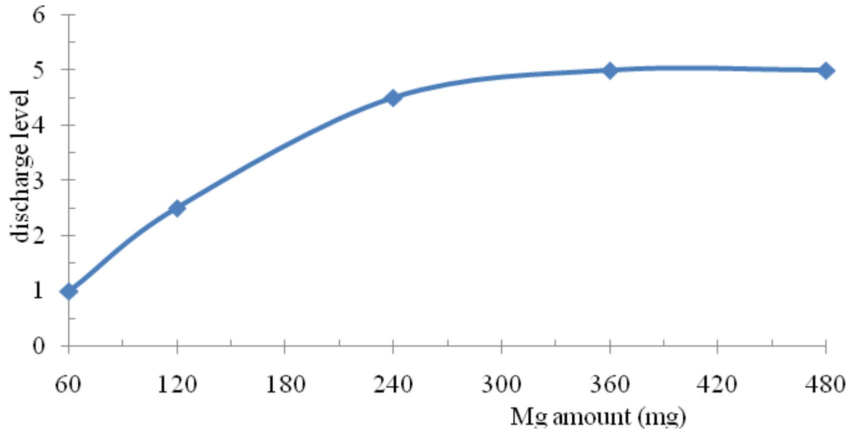

Figure S5. Correlation of metal amount with discharge level (Table 3). For experimental conditions, see Figure S4.

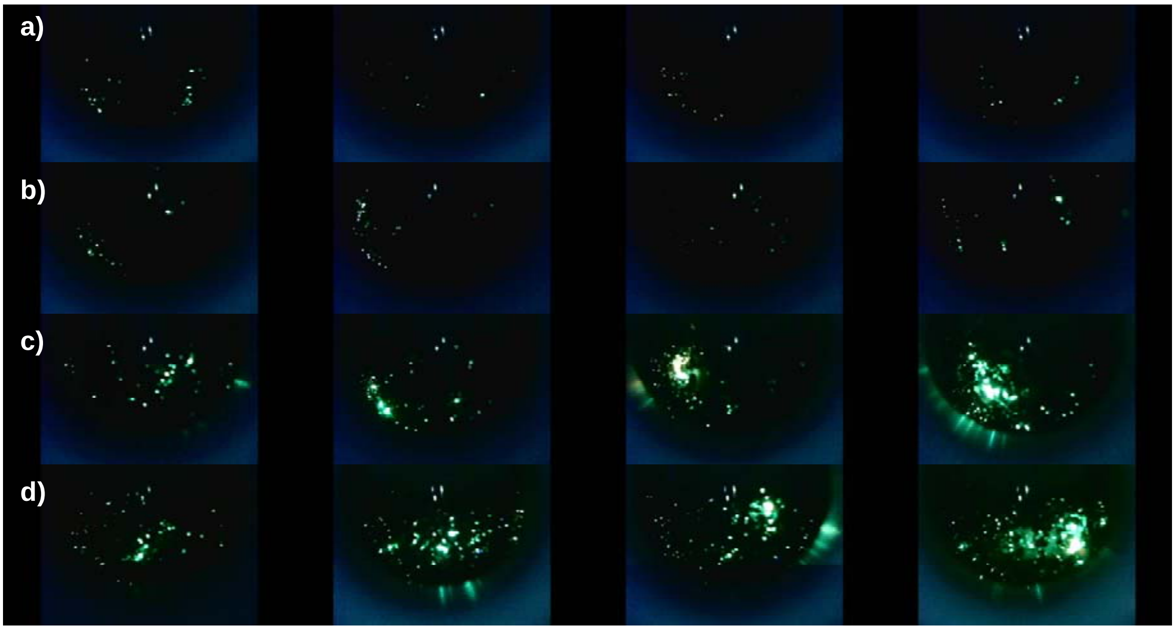

Figure S6. Effect of Mg particle size (300 W constant power, 120 mg Mg, 3 mL benzene, Particles 1.1 to 1.4 in Table S1) on electrical discharges. (a) 250-400µm, (b) 400-500 µm, (c) 500–630 µm, (d) 630-800 µm. See also Table S2. Irradiation experiments were performed for 30 s. Shown are still images of the videos obtained with the build-in camera.

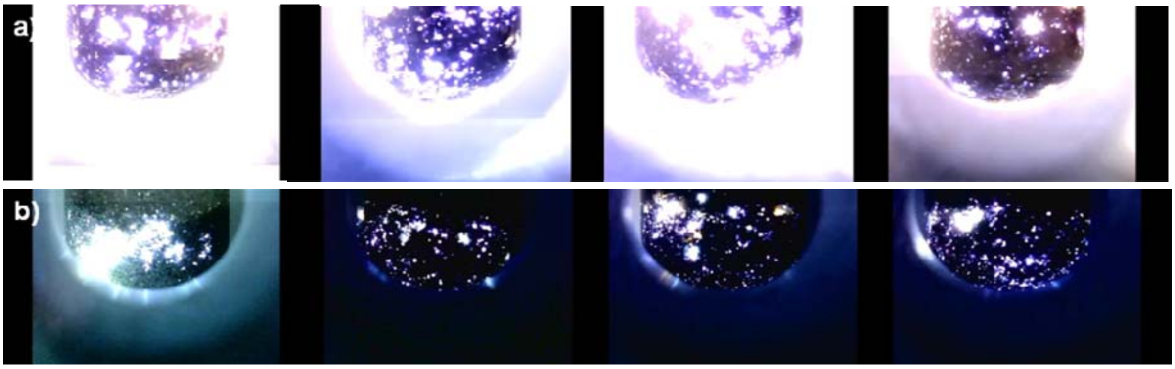

Figure S7. Comparison of electrical discharges with (a) Zn granules/needles (Particles 2.2) and (b) spherical Zn powder (Particles 1.5) (Table S4) in benzene (300 W constant power, particle size 180-250 µm, 3 mL benzene). Shown are still images of the videos obtained with the built-in camera (time increases from left to right).

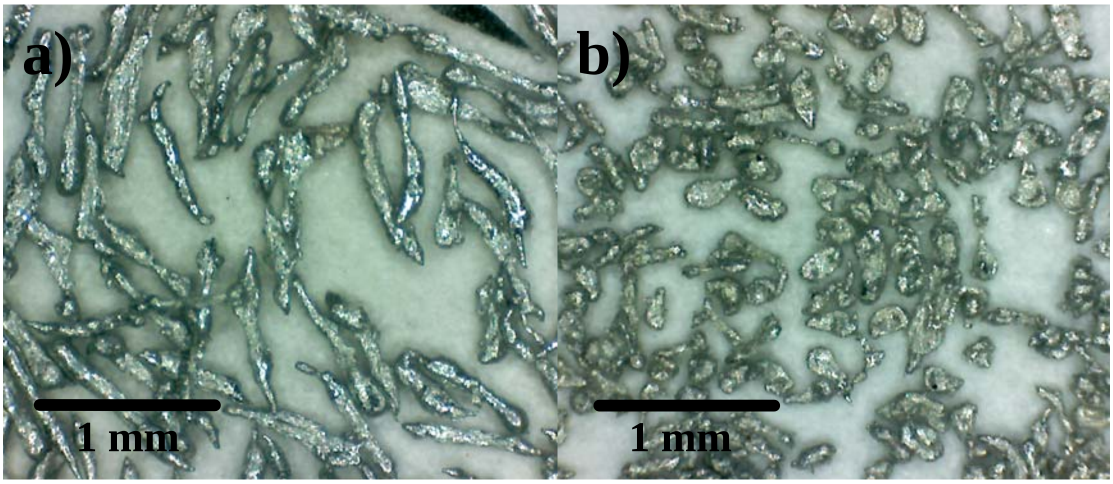

Figure S8. Microscopy images of (a) Zn granules (needles, Particles 2.1**)** and (b) Zn powder (spherical, Particles 1.4) (Table S4).

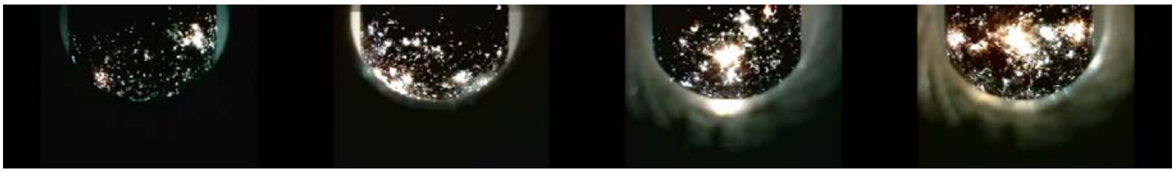

Figure S9. Electrical discharges with 618 mg Cu particles 1.1 (Table S5) in benzene (300 W constant power, particle size 125-180 µm, 3 mL benzene). Shown are still images of the videos obtained with the build-in camera.

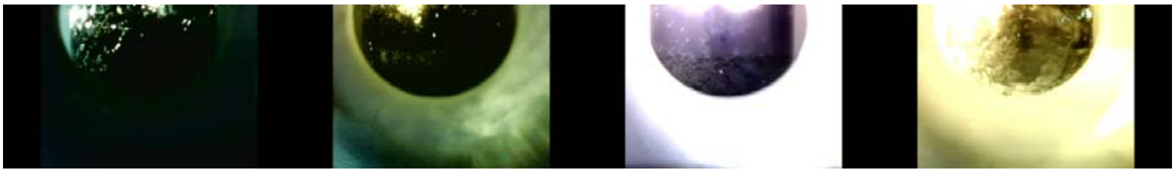

Figure S10. Electrical discharges with 542 mg Fe particles 2.4 (Table S7) in benzene (300 W constant power, particle size 125-180 µm, 3 mL benzene). Shown are still images of the videos obtained with the built-in camera.

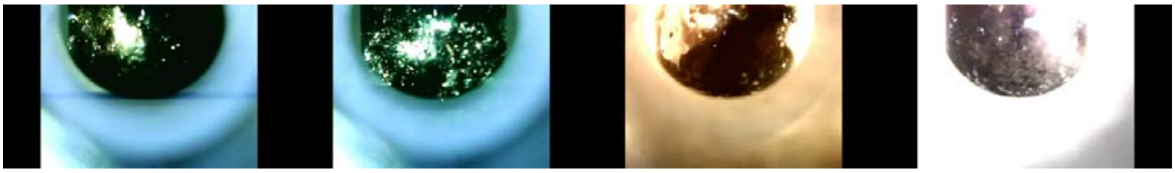

Figure S11. Electrical discharges with 615 mg Ni particles 1.3 (Table S9) in benzene (300 W constant power, particle size 125-180 µm, 3 mL benzene). Shown are still images of the videos obtained with the build-in camera.

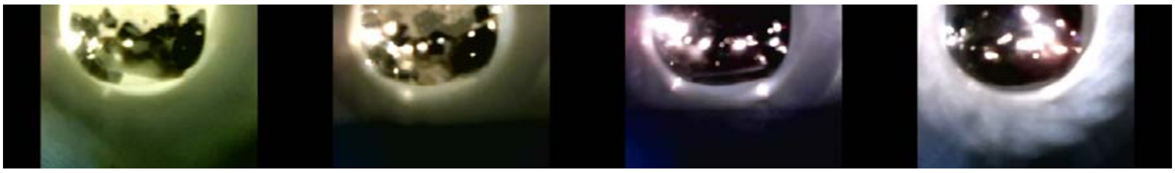

Figure S12. Electrical discharges with 219 mg of SiC granules in benzene (300 W constant power, particle size 1500-2500 µm, 3 mL benzene). Shown are still images of the videos obtained with the build-in camera.

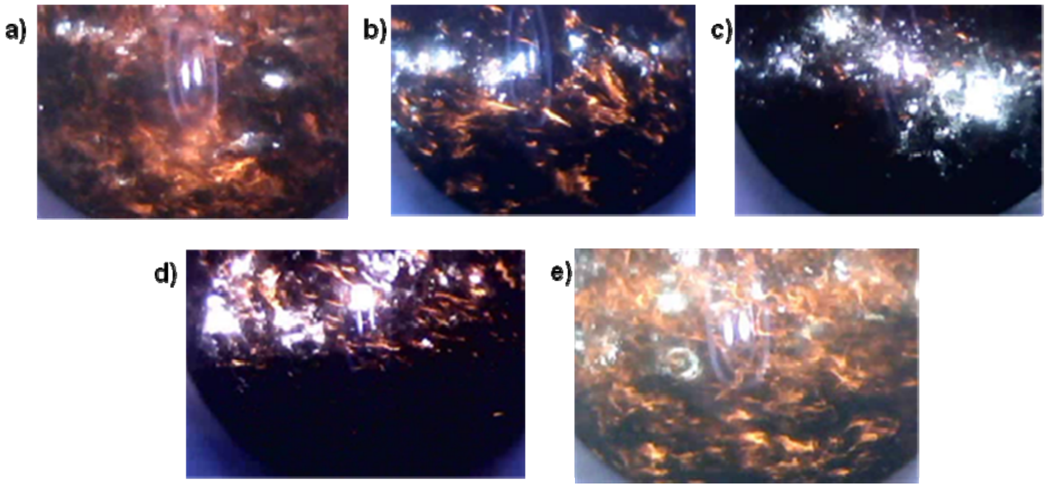

Figure S13. Electrostatic discharges (arcing) of metal and metal oxide powders (300 mg) suspended in decaline (Monowave 300, 250 °C): (a) Fe (Sigma-Aldrich, cat. No. C3518-100g, Lot: 115K0724, average particle size 5 µm), (b) Fe (Aldrich, cat. no. 20.930-9, Lot: U21678-278, average particle size 44 µm), (c) Fe (Acros Organics, cat. no. 197811000, Lot: A0277460, average particle size 212 µm), (d) Fe_3O_4 (Aldrich, cat. no. 518158-10g, Lot: MKBC3159V), and (e) Ni (Sigma-Aldrich, cat. no. 203904, Lot: 01109KE, average particle size 150 µm).

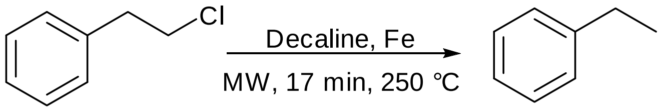

Scheme S1**.** Reductive dehalogenation of (2-chloroethyl)benzene with Fe^{0} under microwave conditions.[S2]

References:

[S1] B. Gutmann, A. M. Schwan, B. Reichart, C. Gspan, F. Hofer, C. O. Kappe, *Angew. Chem., Int. Ed.* **2011**, *50*, 7636.

[S2] Y. Tsukahara, A. Higashi, T. Yamauchi, T. Nakamura, M. Yasuda, A. Baba, Y. Wada, *J. Chem. Phys. C* **2010**, *114*, 8965.
